# Epigenetic Dysregulation of Mammalian Male Meiosis Caused by Interference of Recombination and Synapsis

**DOI:** 10.3390/cells10092311

**Published:** 2021-09-03

**Authors:** Roberto de la Fuente, Florencia Pratto, Abrahan Hernández-Hernández, Marcia Manterola, Pablo López-Jiménez, Rocío Gómez, Alberto Viera, María Teresa Parra, Anna Kouznetsova, R. Daniel Camerini-Otero, Jesús Page

**Affiliations:** 1 Department of Biology, Faculty of Sciences, University Autonoma of Madrid, 28049 Madrid, Spain; r.delafuente@igbzpan.pl (R.d.l.F.); pablo.lopezj@uam.es (P.L.-J.); rocio.gomez@uam.es (R.G.); alberto.viera@uam.es (A.V.); mayte.parra@uam.es (M.T.P.); 2 Department of Experimental Embryology, Institute of Genetics and Animal Biotechnology of the Polish Academy of Sciences, Jastrzębiec, 05-552 Magdalenka, Poland; 3 Genetics and Biochemistry Branch, National Institute of Diabetes and Digestive and Kidney Diseases, National Institutes of Health, Bethesda, MA 20892, USA; florencia.pratto@nih.gov (F.P.); rdcamerini@mail.nih.gov (R.D.C.-O.); 4 Department of Cell and Molecular Biology, Karolinska Institute, 171 77 Stockholm, Sweden; abrahan.h.hernandez@gmail.com (A.H.-H.); anna.kouznetsova@ki.se (A.K.); 5 Department of Human Genetics, Institute of Biomedical Sciences, Faculty of Medicine, University of Chile, Santiago 8380453, Chile; mmanterola@uchile.cl

**Keywords:** meiosis, epigenetics, histone modifications, synapsis, recombination

## Abstract

Meiosis involves a series of specific chromosome events, namely homologous synapsis, recombination, and segregation. Disruption of either recombination or synapsis in mammals results in the interruption of meiosis progression during the first meiotic prophase. This is usually accompanied by a defective transcriptional inactivation of the X and Y chromosomes, which triggers a meiosis breakdown in many mutant models. However, epigenetic changes and transcriptional regulation are also expected to affect autosomes. In this work, we studied the dynamics of epigenetic markers related to chromatin silencing, transcriptional regulation, and meiotic sex chromosome inactivation throughout meiosis in knockout mice for genes encoding for recombination proteins SPO11, DMC1, HOP2 and MLH1, and the synaptonemal complex proteins SYCP1 and SYCP3. These models are defective in recombination and/or synapsis and promote apoptosis at different stages of progression. Our results indicate that impairment of recombination and synapsis alter the dynamics and localization pattern of epigenetic marks, as well as the transcriptional regulation of both autosomes and sex chromosomes throughout prophase-I progression. We also observed that the morphological progression of spermatocytes throughout meiosis and the dynamics of epigenetic marks are processes that can be desynchronized upon synapsis or recombination alteration. Moreover, we detected an overlap of early and late epigenetic signatures in most mutants, indicating that the normal epigenetic transitions are disrupted. This can alter the transcriptional shift that occurs in spermatocytes in mid prophase-I and suggest that the epigenetic regulation of sex chromosomes, but also of autosomes, is an important factor in the impairment of meiosis progression in mammals.

## 1. Introduction

During the prophase of the first meiotic division, homologous chromosomes undergo a series of distinctive processes: pairing, synapsis, and recombination. The occurrence of these events is dependent on cytological and molecular mechanisms that have been intensively studied in the last few decades [1]. Homologous chromosomes start to pair at leptotene and initiate synapsis during zygotene, which involves the assembly of a proteinaceous structure called synaptonemal complex (SC) [2,3]. The SC comprises two lateral elements (LEs), one per homologous chromosome, and a series of transverse filaments (TFs) that bridge between the LEs and assemble in the center of the SC, forming the central element (CE) [4,5,6]. In mammals, the LEs are formed by proteins SYCP3 and SYCP2, while TFs are composed by the protein SYCP1 [7,8,9]. Synapsis between homologues is complete during pachytene and at diplotene it is dissolved when SYCP1 detaches from the LEs, allowing homologous chromosomes to separate. Simultaneously to pairing and synapsis, homologous chromosomes undergo meiotic recombination. In organism such as yeast, plants, and mammals, recombination is initiated during leptotene with the induction of hundreds of DNA double strand breaks (DSBs). This requires the action of the SPO11–TOP6BL transesterase and a number of accessory proteins, including the members of the MRN complex (composed by MRE11, RAD51, and NBS1 in mammals), which contributes to the processing of DSBs, and the RMM complex (REC114, MEI4, and IHO1 in mammals), which associate with the SC and promote DSBs [10,11]. Subsequently, a DNA damage response (DDR) is triggered, activating the homologous recombination repair pathway. This requires the action of several proteins, including the recombinases RAD51 and DMC1. These proteins, in collaboration with additional partners such as HOP2, promote the association of broken DNA molecules with an intact template on the homologous chromosome to initiate DNA repair [12,13,14]. The interactions that initiate chromosomal contacts to repair DNA are the basis for homologous recognition and synapsis during zygotene in most species, although in *Drosophila* and *Caenorhabditis elegans* both pairing and synapsis rely on alternative mechanisms [15,16]. As zygotene and pachytene proceed, these DSBs are repaired, mainly yielding gene conversion events between homologues. Only a small fraction of these DSBs is processed to produce reciprocal recombination events, which eventually lead to the formation of connections between homologous chromosomes called crossovers (CO). The action of MLH1 and MLH3 proteins is required for the final stages of CO formation [17,18,19]. The cytological manifestations of COs are called chiasmata, which maintain the association of homologous chromosomes from diplotene, when the SC is disassembled, until anaphase-I, when they segregate to opposite cell poles.

While these events constitute the main core of chromosome behavior during the first meiotic prophase, chromatin organization, composition, and activity have also emerged as key processes that regulate meiosis in mammals. First, the organization of chromatin undergoes changes during first meiotic prophase. In mammals, recent studies have revealed that the interphase chromatin organization in topologically associated domains (TADs) is completely rearranged during prophase-I [20,21,22]. Both intra- and interchromosomal contacts are mainly lost and then replaced by a different interaction pattern reflecting the synapsis of homologous chromosomes. Only the specific association of heterochromatic regions of centromeres remain as notable interchromosomal interactions [23].

Second, prophase-I is unique for representing a period of high transcriptional activity [24,25]. Contrary to mitotic prophase, first meiotic prophase is characterized by the expression of a large set of genes [26]. Moreover, both the bulk of transcription activity and the gene expression profile change throughout different prophase-I substages [27,28,29,30]. Thus, the transcriptional profile typical of the spermatogonia is replaced at the beginning of meiosis by a different one, which involves the specific expression of genes required for synapsis and recombination. Then, from pachytene onwards, spermatocytes start to actively express genes required for meiosis and spermiogenesis [27]. These expression profiles are accompanied by changes in the overall transcription activity, which is relatively low during leptotene and zygotene and burst during mid pachytene up to the end of diplotene [25,30,31].

Third, concomitantly with the changes in transcription activity, chromatin undergoes a series of modifications during prophase-I that involves changes in both DNA and histones. Several histone variants have been shown to have a specific loading cycle during meiosis. The best characterized is the incorporation of H1t, which replaces canonical H1 in autosomes from mid pachytene onwards [32,33] and may facilitate an open chromatin configuration [34]. In addition, multiple histone modifications, including phosphorylation, methylation, acetylation, ubiquitination and others, occur during meiosis in a stage-dependent manner [35,36,37]. Some of these modifications can be related to the regulation of chromatin conformation, such as acetylation of H3 at lysine 9 and trimethylation of H3 at lysine 9, which are involved in generating open and closed chromatin configurations, respectively. Other histone modifications have been specifically related to exclusive meiosis processes. This is the case for histone H3 trimethylation at lysine 4 (H3K4me3) by the methyltransferase PRDM9, which marks the sites for DSB production by SPO11 at the beginning of meiosis [38,39,40,41]. Likewise, the phosphorylation of H2AX (giving rise to γH2AX) is an essential step to trigger DNA repair in response to DSBs generation [42]. Consistent with the importance of these epigenetic modifications for the outcome of meiosis, mutations affecting the enzymes that catalyze histone modifications cause meiotic arrest and sterility [43,44,45,46,47].

One of the most studied chromatin modification events in mammalian meiosis is related to sex chromosomes. X and Y chromosomes in male mammals are strikingly different in size and gene content and only share a small terminal region of homology called pseudoautosomal region (PAR) [48]. These differences cause that during pachytene sex chromosomes remain unsynapsed for most of their length [49]. Extensive asynapsis triggers a specific epigenetic response leading to the compaction of sex chromosomes to form a distinctive chromatin compartment called the sex body. This response includes the accumulation of specific proteins (such as SUMO-1, ASYNAPTIN, XMR, XY77, and ATR), histone variants (H3.3) and histone modifications (most importantly γH2AX), resulting in a transcriptional inactivation process called meiotic sex chromosome inactivation (MSCI) [50,51]. MSCI has been proposed to be an important event for the regulation of meiosis progression in male mammals, as interference of MSCI can allow the expression of *Zfy1/2* genes present in the Y chromosome [52], causing an interruption of meiosis progression, spermatocyte apoptosis and male infertility [53]. According to this hypothesis, many reports have highlighted the fact that sex chromosome inactivation is absent in mutants for genes involved in synapsis, recombination or sister chromatid cohesion [54,55,56,57,58,59]. Furthermore, in most of these mutant mice meiosis is arrested at a similar stage, which has been identified as the stage IV of the seminiferous tubule [54,60].

Failure to trigger MSCI has been considered key to produce meiosis breakdown in many meiotic mutants. However, the complexity of the epigenetic and gene expression landscapes in prophase-I spermatocytes raised the question as to whether additional genome-wide epigenetic factors may be also dysregulated in those cases [31,54,61,62]. Indeed, many meiotic mutants with defects in early recombination events or chromosome synapsis also fail to achieve some whole-nucleus epigenetic transitions, most typically the incorporation of histone H1t [54,57]. Therefore, the study of the factors affecting the epigenetic transitions of the whole nucleus, with a potential influence on transcriptional levels and gene expression profiles, seems to be crucial to understand the regulation of meiosis progression.

Here we aimed to explore the alterations of the normal epigenetic program when meiosis progression is disturbed by defects in synapsis or recombination. We present the localization patterns of epigenetic markers that appear during meiosis and that seem to be related to transcription regulation, MSCI, and pachytene progression. We have studied their dynamics in mice knockout for *Spo11*, *Dmc1*, *Hop2*, *Mlh1*, *Sycp3*, and *Sycp1*, which represent some of the best characterized models of meiosis disruption, present a variety of recombination and synapsis defects and are widely used in meiotic studies [17,18,57,63,64,65,66]. Our results show that the epigenetic profile reached in each mutant is not directly correlated to the morphological progression of synapsis or recombination, and that spermatocytes may simultaneously show both early and late epigenetic features. This shows the potential for uncoupling the functional modification of chromatin and the progression of meiosis, and also reveals an unreported epigenetic desynchronization.

## 2. Materials and Methods

### 2.1. Ethical Approval

All experimental procedures with animals were approved by local organisms and appropriate methods were applied. Procedures in Spain were performed according to the RD120172005 on Protection of Animals for Research and Other Scientific Goals (21 October 2005). Approval was provided by the Institutional Animal Care and Use Committee in USA, the Stockholm-North Animal Ethical Committee in Sweden (application number 181/09) and Universidad Autónoma de Madrid (Ethics Committee Certificate CEI 55-999-A045).

### 2.2. Mouse Genotypes

*Spo11*^-/-^, *Dmc1*^-/-^, *Hop2*^-/-^ and *Mlh1*^-/-^ mice have been described previously [18,64,65,67]. Lines with *Sycp1*^-/-^ and *Sycp3*^-/-^ genotypes were obtained as described [57,66]. At least two individuals were analyzed per genotype.

### 2.3. Spermatocyte Preparation

Testes from adult males were dissected and the tunica albuginea removed. Seminiferous tubules were placed in phosphate buffered saline (PBS: 137 mM NaCl, 2.7 mM KCl, 10.1 mM Na_2_HPO_4_, 1.7 mM KH_2_PO_4_, pH 7.4) and subsequently processed for spreading techniques following the drying-down technique described previously [68], with slight modifications. Tubules were disaggregated in PBS and chopped with a razor in a Petri dish to obtain a cell suspension. 400 μL of 100 mM sucrose was then added to this cell suspension and cells were decanted for 2 min and subsequently spread onto a slide submerged in 1% formaldehyde-containing distilled water with 50 mM Na_2_B_4_O_7_ and 0.15% Triton X-100. After air-drying, slides were washed with 0.04% Photo-Flo (Kodak) in distilled water and air-dried before use for immunofluorescence.

### 2.4. Immunofluorescence

Following rehydration in PBS for 10 min, slides were first blocked with goat serum in PBS for 1 h. Then, incubation with primary antibodies was carried out overnight at 4 °C in a moist chamber upon the following dilutions in PBS: mouse monoclonal anti-SYCP3 (Ab97672, Abcam, Cambridge, UK) at a 1:200 dilution; rabbit anti-SYCP3 (Ab15093, Abcam) at a 1:100 dilution; guinea pig anti-STAG3 raised as described [69] at 1:100; mouse monoclonal against histone H2AX phosphorylated at serine 139 (γH2AX) (#05-636, Millipore, MA, USA) at a 1:1000 dilution; mouse anti-RNA polymerase II phosphorylated at serine 2 (Ab24758, Abcam) at a 1:100 dilution; rabbit anti-histone H3 trimethylated at lysine 9 (H3K9me3) (Ab8898, Abcam) at a 1:100 dilution; rabbit anti-histone H3 monomethylated at lysine 4 (H3K4me1) (Ab8895, Abcam) at a 1:100 dilution; mouse anti-histone H3 acetylated at lysine 9 (H3K9ac) (H 0913, Sigma, St. Louis, MO, USA); mouse anti-GMP-1 (SUMO-1) (33-2400, ThermoFisher Scientific, Rockford, IL, USA) at a 1:100 dilution; and guinea pig anti H1t (a gift from Dr. Mary Ann Handel) at a 1:250 dilution. Slides were then rinsed in PBS 3 × 5 min each and incubated with appropriate secondary antibodies in a moist chamber at room temperature for 1 h. We used anti-rabbit, anti-mouse and anti-guinea pig secondary antibodies raised in donkey and anti-guinea pig secondary antibodies raised in goat. Antibodies were conjugated either with Alexa 350, Alexa 488, Alexa 594 (Invitrogen, Eugene, OR, USA), Cy3 or DyLight 649 (Jackson ImmunoResearch Laboratories, West Grove, PA, USA). Slides were subsequently rinsed in PBS 3 × 5 min each, and either stained 3 min with 10 μg/mL DAPI and mounted with Vectashield (Vector Laboratories, Burlingame, CA, USA), or directly mounted with DAPI-containing Prolong Gold (Molecular Probes, ThermoFisher Scientific, Rockford, IL, USA). If the two antibodies in the same preparation were raised in the same species, we proceeded as previously described [70].

Observations were made on an Olympus BX61 microscope equipped with a motorized Z axis and images captured with an Olympus DP72 digital camera using the CellF software (Olympus, Hamburg, Germany), and on a Leica DMRA2 microscope capturing images with a Hamamatsu digital CCD camera C4742-95 and Openlab 3.1.4 software. Image processing was made using ImageJ (National Institutes of Health, Bethesda, MD, USA) and Adobe Photoshop 7.0 software (Adobe Systems Inc., CA, USA).

## 3. Results

To analyze the epigenetic consequences produced when DNA recombination and synapsis are interfered in meiosis, we studied *Spo11*^-/-^, *Dmc1*^-/-^, *Hop2*^-/-^ and *Mlh1*^-/-^ mice, which present alterations at different steps of the recombination pathway, and *Sycp1*^-/-^ and *Sycp3*^-/-^ mice, which are defective in the assembly of the axial/lateral elements (AEs/LEs) and the transverse filaments (TFs) of the SC, respectively. For all these genotypes, we studied the localization pattern of the same markers: H3K9me3 and H3K4me1, which distribute widely in the nucleus at early prophase-I and are related to transcription downregulation [31,37]; γH2AX as a marker of DNA damage and MSCI [42,71]; SUMO1, as a protein that is incorporated early to sex chromosomes upon MSCI initiation [72]; RNA polymerase II phosphorylated at serine 2 (pRNA pol-II); H3K9ac that is associated to open chromatin configuration and transcription activity [73]; and H1t, as a marker widely used to assess pachytene progression past the stage IV of the seminiferous epithelium [32,33,54].

**Figure 1:**
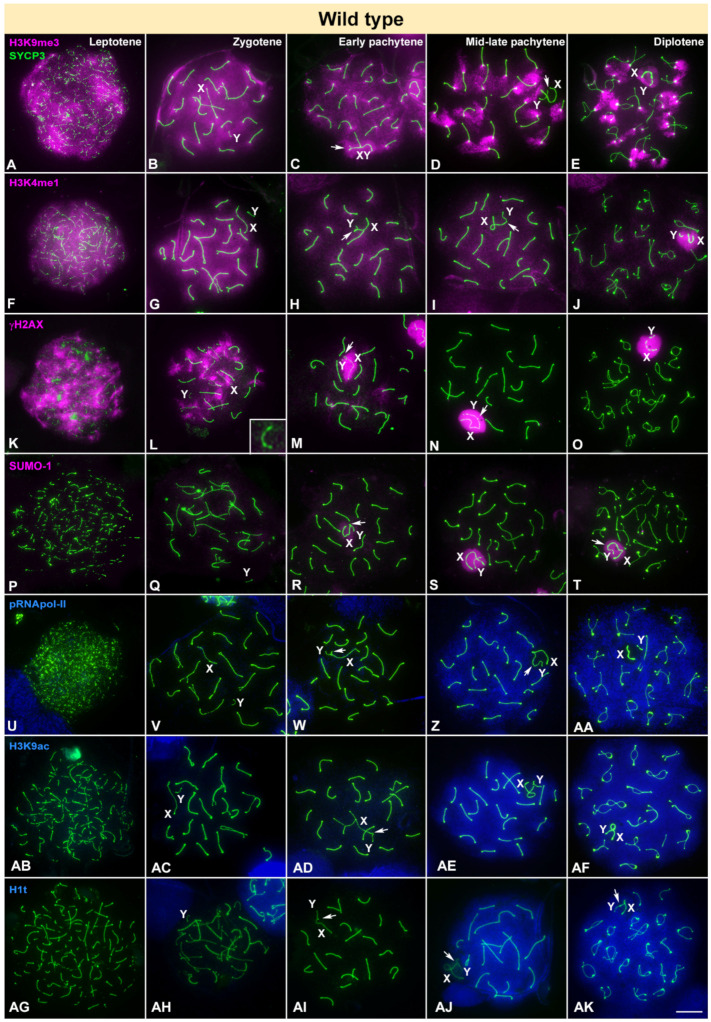
Immunolocalization of SYCP3 (green) and epigenetic markers of silencing (pink) or transcription activation (blue) in wild type mice. X, Y indicate sex chromosomes. Arrow indicates the synapsed region of sex chromosomes. (**A**–**E**). The heterochromatin-related factor H3K9me3 is distributed in the whole nucleus from leptotene (**A**) to zygotene (**B**), including the Y chromosome. Then at early pachytene (**C**) H3K9me3 decreases in autosomes but remains in the sex chromosomes (XY) and the pericentromeric regions. During mid-late pachytene (**D**) it is present only in the pericentromeric regions, but not the sex chromosomes (XY). At diplotene (**E**) the protein is mainly restricted to the pericentromeric regions. Sex chromosomes (XY) become labelled again. (**F**–**J**). H3K4me1 is strongly detected in leptotene (**F**) and zygotene (**G**) spermatocytes. Labelling is weaker in early (**H**) and mid-late pachytene (**I**) spermatocytes. (**J**) At diplotene sex chromosomes (XY) are intensely marked. (**K**–**O**). γH2AX signal covers the whole nucleus during leptotene (**K**) and decreases at zygotene (**L**). The Y chromosome is initially devoid of γH2AX (inset). (**M**). The sex pair is intensely marked at early pachytene with γH2AX and remains so in mid-late pachytene (**N**) and diplotene (**O**). (**P**–**T**). No labelling of SUMO-1 is detected in leptotene (**P**) or zygotene (**Q**). A faint signal is detected on the sex chromosomes (XY) at early pachytene (**R**) and increases in intensity in mid-late pachytene (**S**) and diplotene (**T**). (**U**–**AA**). pRNApol-II is not detected during leptotene (**U**), zygotene (**V**) or early pachytene (**W**). At mid-late pachytene (**Z**) and diplotene (**AA**) a strong labelling of pRNApol-II is seen in the entire nucleus with the exception of the XY body (XY). (**AB**–**AD**). H3K9ac is not detected from leptotene to early pachytene. (**AE**–**AF**). In mid-late pachytene and diplotene a strong mark is detected in the nucleus, with the exception of the sex chromosomes (XY). (**AG**–**AK**). Spermatocytes are devoid of H1t labelling from leptotene (**AG**) to early pachytene (**AI**), and an intense labelling is observed from late pachytene (**AJ**) to diplotene (**AK**). Scale bar: 10 µm.

The pattern we have observed for all of these markers in wild type mice (Figure 1) is in agreement with previous reports [31,33]. H3K9me3, a typical marker for heterochromatin and gene silencing [74], is distributed throughout the chromatin during leptotene and zygotene (Figure 1A,B), but starts to fade away from the autosomes in early pachytene, remaining concentrated in the sex chromosomes and in the pericentromeric regions of the autosomes (Figure 1C). By mid-late pachytene this histone variant is removed from the sex chromosomes and it only remains in the pericentromeric regions from all chromosomes except the Y (Figure 1D). At diplotene, H3K9me3 re-accumulates faintly in the sex chromosomes (Figure 1E). Thus, the dynamics of H3K9me3 localization reveals that at the beginning of meiosis the chromatin presents a conspicuous accumulation of an epigenetic factor involved in transcription downregulation that is subsequently removed as prophase-I progresses. Correspondingly, we observed that H3K4me1, which has been also related to chromatin silencing [75], is intensely detected throughout almost all chromatin during leptotene and zygotene (Figure 1F,G). At early pachytene (Figure 1H), this histone modification usually covers the whole nucleus, but is gradually removed and remains barely detectable until late pachytene (Figure 1I). At diplotene, H3K4me1 intensely accumulates over the sex chromosomes (Figure 1J).

γH2AX appears as large foci distributed at leptotene, covering most of the nucleus (Figure 1K). At zygotene, with synapsis progression, it starts to be removed from the synapsed chromosomal regions while remaining in those still unsynapsed (Figure 1L). The Y chromosome, which can be identified at late zygotene by a typical thickening of the PAR-bearing end, is usually devoid of labelling at this stage. During early pachytene (Figure 1M), γH2AX strongly accumulates in the sex chromosomes, which have already initiated synapsis, and also as small foci that emanate from the SC in some autosomal bivalents. During late pachytene (Figure 1N) and diplotene (Figure 1O) this histone modification remains mostly associated to sex chromosomes, as it is one of the typical marks of MSCI. The formation of a typical sex body and MSCI was corroborated by the presence of SUMO-1. This small-modifying protein is not detected in the nucleus during leptotene or zygotene (Figure 1P,Q). At early pachytene (Figure 1R) a faint signal is observed over the sex chromosomes that becomes more intense at mid-late pachytene (Figure 1S) and diplotene (Figure 1T).

Finally, the two markers of transcription activity, pRNA pol-II and H3K9ac, show a similar pattern to each other, and opposite to the histone modifications aforementioned. Both are barely detectable in the nucleus from leptotene to early pachytene (Figure 1U–W,AB–AD), but increase in mid-late pachytene, intensely covering the whole nucleus with the exception of the sex chromosomes (Figure 1Z,AA,AE,AF). As expected, late pachytene and diplotene spermatocytes incorporate H1t histone (Figure 1AG–AK).

### 3.1. Epigenetic Markers in Recombination-Defective Mutants

**Figure 2:**
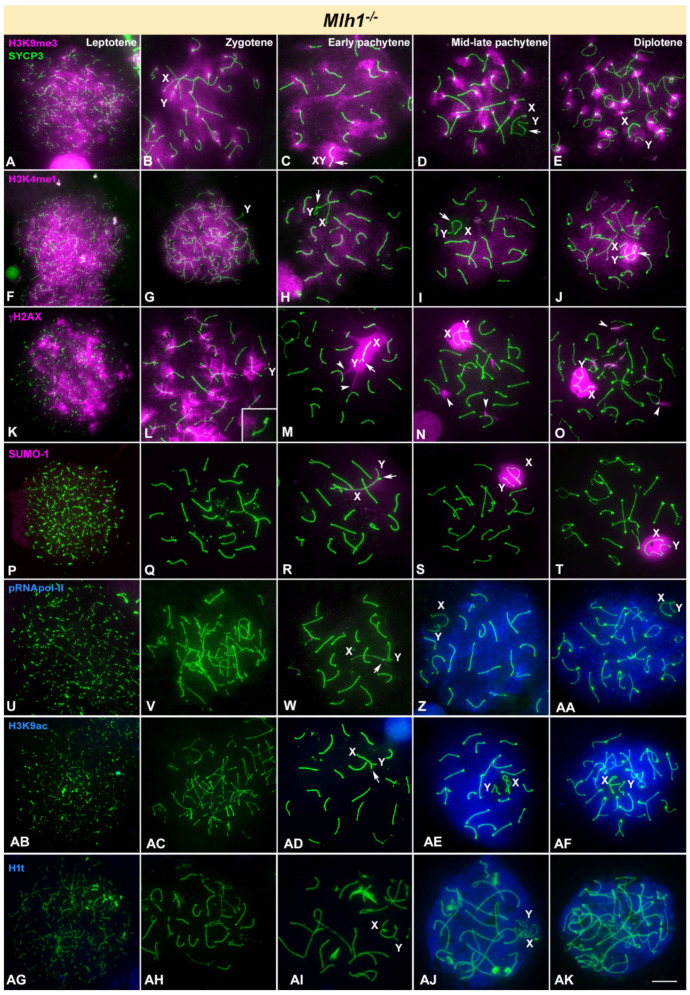
Immunolocalization of SYCP3 (green) and epigenetic markers of silencing (pink) or transcription activation (blue) in *Mlh1* knockout mice. X, Y indicate sex chromosomes. Arrow indicates the synapsed region of sex chromosomes. (**A**–**E**). H3K9me3 is distributed in the whole nucleus from leptotene (**A**). At zygotene (**B**), it is still distributed in all chromosomes, including the X and Y (identified by the distal thickening of the AE) (enlarged in the inset) and slightly accumulates in the pericentromeric regions. (**C**). Early pachytene. H3K9me3 decreases in the autosomes and intensifies in the sex chromosomes (XY) (which appear completely synapsed, arrow) and the pericentromeric heterochromatin. (**D**–**E**). From mid pachytene to diplotene this protein is mainly restricted to the pericentromeric regions. Sex chromosomes (XY) do not show any labelling at late pachytene, excepting the centromeric region of the X chromosome, which shows a weaker labelling. At diplotene, sex chromosomes (XY) become labelled again. (**F**–**J**). H3K4me1 is strongly detected in leptotene (**F**) and zygotene (**G**) in the whole nucleus. A weaker labelling is seen in early (**H**) and mid-late pachytene (**I**) spermatocytes. Sex chromosomes (XY) are partially or completely devoid of labelling. (**J**). At diplotene, autosomes retain a weak H3K4me1 labelling but sex chromosomes (XY) are intensely marked, with the exception of the X centromere. (**K**–**O**). γH2AX signal covers the whole nucleus during leptotene (**K**). At zygotene (**L**), the signal decreases in those chromosomes that have achieved synapsis. The Y chromosome shows no γH2AX labelling (inset). (**M**). At early pachytene the sex pair (XY) is intensely marked with γH2AX. The irregular periphery of the sex chromatin visibly contacts some bivalents that also present discrete regions marked with γH2AX (arrowheads). (**N**). By mid-late pachytene some bivalents still present small γH2AX foci (arrowheads). The sex body presents (XY) a well-defined periphery. (**O**). Diplotene. Sex chromosomes (XY) are intensely labelled, while some foci are still present in some autosomes (arrowheads). (**P**–**T**). No labelling of SUMO-1 is detected in leptotene (**P**) or zygotene (**Q**). Only a very faint signal on the sex chromosomes (XY) is detected at early pachytene (**R**). By mid pachytene (**S**) the intensity of the signal on the sex chromosomes has visibly increased and it is maintained at diplotene (**T**). No other signal is detected on the nucleus at any stage. (**U**–**AA**). pRNApol-II is not detected during leptotene (**U**), zygotene (**V**) or early pachytene (**W**). At mid-late pachytene (**Z**) a strong labelling of pRNApol-II is seen in the entire nucleus with the exception of the XY body. Diplotene spermatocytes (**AA**) maintain the same pattern of localization of pRNApol-II. (**AB**–**AD**). There is no obvious mark of H3K9ac from leptotene to early pachytene. (**AE**). By mid-late pachytene a strong mark for this protein is detected in the nucleus, with the exception of the sex chromosomes (XY). Pericentromeric regions seem to show a less intense signal for this marker. (**AF**). The same pattern can be observed in diplotene spermatocytes. (**AG**–**AK**). The pattern of H1t is identical to wild type: spermatocytes are devoid of H1t labelling from leptotene (**AG**) to early pachytene (**AI**), and then an intense labelling is observed from mid-late pachytene (**AJ**) to diplotene (**AK**). Scale bar: 10 µm.

Among all knockout models studied, *Mlh1*^-/-^ mice exhibit a wild type prophase-I progression. MHL1 protein is involved in the late repair of DSBs to promote crossover formation. Thus, mice lacking this protein show complete and stable synapsis and DNA repair progresses normally until the moment in which COs must be resolved [17,18]. Accordingly, spermatocyte progression is normal up to diplotene (Figure 2) and then stops at metaphase-I when all chromosomes appear as univalents [17]. During prophase-I we only observed subtle synapsis differences compared to wild type in sex chromosome behavior, whose axial elements (AEs) appear frequently detached from each other (even at the PAR) at late pachytene (Figure 2). However, sex chromosomes morphology, which is a usual indicator of meiosis progression [31,76,77] does not show differences compared to wild type mice. The patterns observed for all the epigenetic marks studied are almost identical to those reported in wild type mice (Figure 2). The only clear difference is the persistence of some γH2AX foci associated to autosomes at late pachytene and diplotene in *Mlh1*^-/-^ mice (Figure 2O).

**Figure 3:**
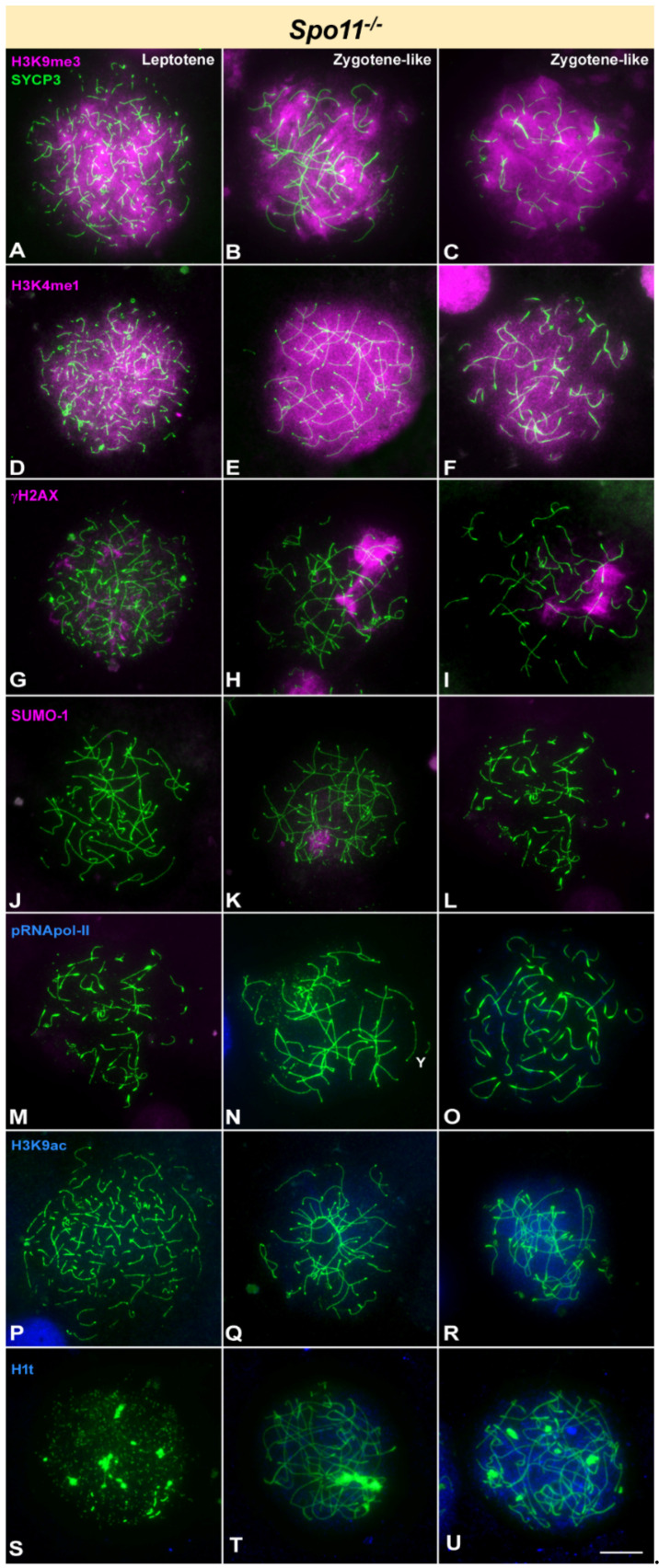
Immunolocalization of SYCP3 (green) and epigenetic markers of silencing (pink) or transcription activation (blue) in *Spo11* knockout mice. Y indicates the Y chromosome, which is identified by its size and morphology of the AE. Cells in each column are classified as leptotene (with no signs of synapsis) and zygotene-like (with different degrees of synapsis). Differential degrees of modifications in the AEs or intensity in the labelling of epigenetic marks are observed in zygotene-like, but spermatocytes are not ascribed to early or late stages. (**A**–**C**). H3K9me3 marks the entire nucleus in all stages detected. Certain chromosome ends show a stronger signal that might correspond to heterochromatin. (**D**–**F**). Although H3K4me1 profusely marks the entire nucleus in leptotene, the intensity of the signal slightly decreases in some zygotene-like stages. (**G**–**I**). γH2AX mark is almost undetectable in the nucleus at leptotene (**G**). However, it is strongly detected on few discrete regions (arrows) in the autosomes in zygotene-like stages (**H**,**I**). (**J**–**L**). SUMO-1 is not detected in leptotene, but some zygotene-like spermatocytes show a faint labelling over a specific region of the nucleus. (**M**–**O**). No detectable signal of pRNApol-II is observed at any stage. (**P**–**R**). H3K9ac is detected as a weak signal only in zygotene-like stages. (**S**–**U**). Leptotene spermatocytes are mostly devoid of H1t, but zygotene-like ones can show an H1t labelling, which is variable in intensity. Scale bar: 10 µm.

We next analyzed the location of the same proteins in *Spo11*^-/-^ mice. The absence of SPO11 in mouse abolishes the production of DSBs, causing synapsis to be only partial and mostly heterologous [63,65]. Thus, cells from *Spo11*^-/-^ mice do not progress normally into prophase-I and enter apoptosis in a zygotene-like stage. Therefore, we catalogued spermatocytes to be just in leptotene or zygotene-like stage owing to the absence or presence of synapsed chromosomes, respectively. Although differences can be found in zygotene-like spermatocytes regarding the morphology of AE/LEs and the extension of synapsis, we could not unambiguously ascertain if they represent different stages. We observed that H3K9me3 and H3K4me1 appear covering most part of the chromatin in leptotene spermatocytes, indicating that induction of these histone modifications is not dependent on DSBs and they are incorporated at the beginning of meiosis in the absence of SPO11 (Figure 3A,D). The intense labelling of these two epigenetic marks is maintained almost unaltered in all zygotene-like spermatocytes (Figure 3B,C,E,F). Regarding γH2AX, the absence of DSBs mostly abolishes the presence of this histone modification, as previously reported [54,55]. Leptotene spermatocytes only show some small and scattered foci (Figure 2G), while in zygotene-like about half of the spermatocytes (48.63%, *n* = 293) accumulate large γH2AX foci over some chromosomes (Figure 3H,I). This large γH2AX focus is usually referred to as pseudo-sex body, even though it is not associated to sex chromosomes [54,55]. SUMO-1 does not accumulate in *Spo11*^-/-^ leptotene spermatocytes (Figure 3J), but 46.96% (*n* = 526) of zygotene-like spermatocytes showed an accumulation of SUMO-1 (Figure 3K,L). This signal co-localizes with γH2AX (Supplementary Figure S1), indicating that the pseudo-sex body may also incorporate other typical markers of MSCI, as previously reported for XMR [54]. Transcription activity marker pRNA pol-II is absent in *Spo11*^-/-^ spermatocytes (Figure 3M–O). However, contrary to wild type and *Mlh1*^-/-^, the absence of pRNA pol-II seems to be uncoupled from H3K9ac, as this histone mark does faintly accumulate in 56.54% of the zygotene-like spermatocytes (*n* = 543) (Figure 3P–R). As previously described [54], *Spo11*^-/-^ spermatocytes incorporate H1t histone (Figure 3S–U). We found that in our *Spo11*^-/-^ samples up to 83.74% (*n* = 535) zygotene-like spermatocytes may show H1t labelling. The intensity of this labelling is very weak in some spermatocytes while in others can reach levels comparable to wild type. Not clear correspondence of this intensity with other features, such as extension of synapsis or modifications of the AEs, could be established.

**Figure 4:**
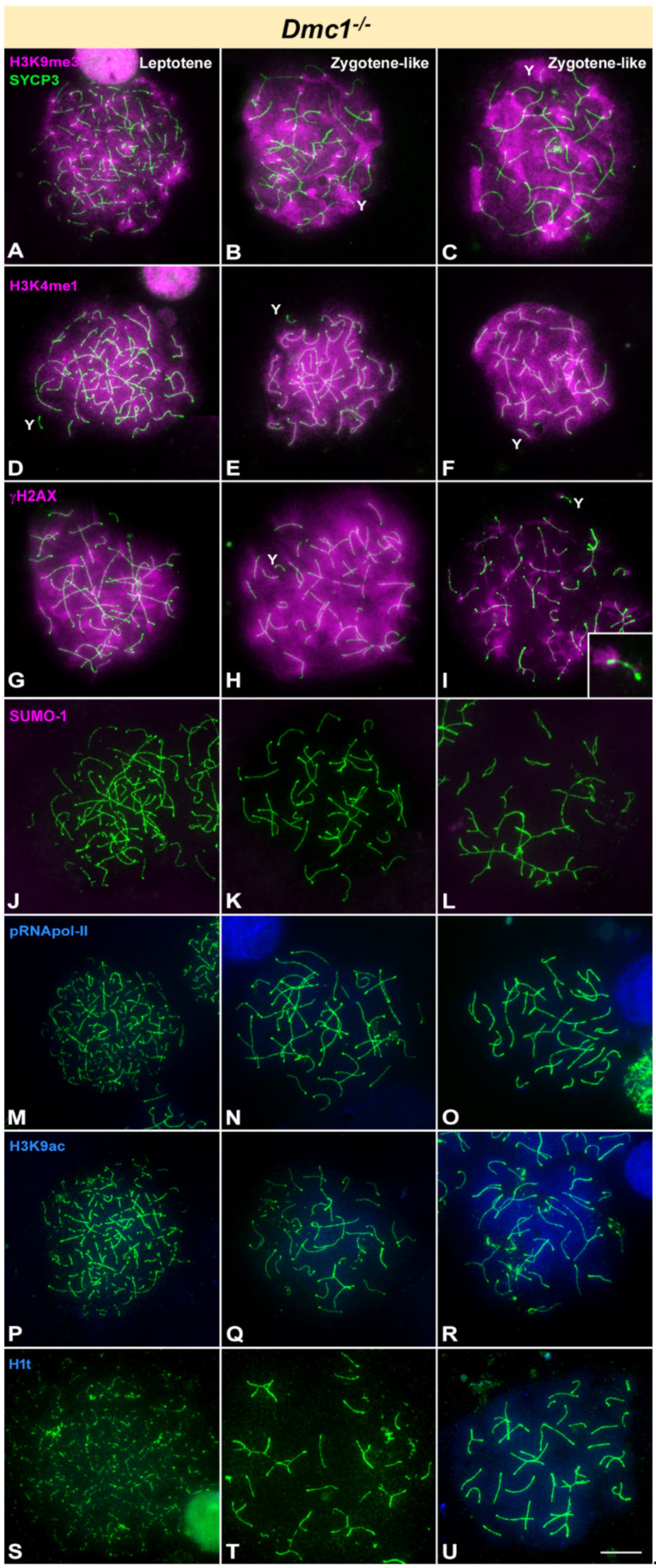
Immunolocalization of SYCP3 (green) and epigenetic markers of silencing (pink) or transcription activation (blue) in *Dmc1* knockout mice. Y indicates the Y chromosome, which is identified by its size and morphology of the AE. Cells in each column are classified as leptotene (with no synapsis) and zygotene-like (with different degrees of synapsis). Differential degrees of modifications in the AEs or intensity in the labelling of epigenetic marks are observed in zygotene-like, but spermatocytes are not ascribed to early or late stages. (**A**–**C**). A prominent mark for H3K9me3 is detected in all stages covering the whole nucleus and slightly more intense in the pericentromeric regions. (**D**–**F**). H3K4me1 strongly marks the entire nucleus of the spermatocytes at all stages. (**G**–**I**). γH2AX labelling is spread over the entire nucleus in leptotene and some zygotene-like spermatocytes. In other zygotene-like stages (**I**) labelling is partially reduced in the autosomes. A single focus is observed in the Y chromosome (inset). (**J**–**L**). SUMO-1 is not detected at any stage in these mutants. (**M**–**O**). No signal of pRNApol-II is detected at any stage. (**P**–**R**). H3K9ac is weakly detected in leptotene and more intensely in some zygotene-like spermatocytes. (**S**–**U**). H1t is usually absent in leptotene spermatocytes. Some zygotene-like spermatocytes can appear mostly devoid of H1t labelling (**T**), while others show an appreciable accumulation of H1t (**U**). Scale bar: 10 µm.

Mammalian DMC1 has a role in the initial steps of recombination, and consequently spermatocytes lacking DMC1 protein arrest in prophase-I with unresolved DSBs and largely unsynapsed chromosomes [12,67]. Similar to *Spo11*^-/-^ mice, we classified *Dmc1*^-/-^ spermatocytes as leptotene or zygotene-like stages. As expected, H3K9me3 is detected in all stages covering the whole nucleus (Figure 4A–C), thus maintaining a distribution in the most advanced stages similar to wild type zygotene. Likewise, H3K4me1 is detected covering the whole nucleus and never fading away (Figure 4D–F). Regarding the distribution of γH2AX, this protein covers the chromatin almost entirely at early stages (Figure 4G) and tends to disappear as synaptic associations take place between chromosomes at zygotene-like stage (Figure 4H,I). In the spermatocytes in which γH2AX labelling is no longer spread all over the nucleus, the Y chromosome can be discerned displaying a γH2AX focus, presumably at the PAR. However, there is neither formation of a sex body nor signs of MSCI, which is corroborated by the complete absence of SUMO-1 (Figure 4J–L). Our observations are consistent with low levels of transcription in all stages since pRNA pol-II is not detected at any time (Figure 3M–O). Again, H3K9ac cycle is desynchronized with pRNA pol-II, and this histone modification is found, though faintly, in 53.57% of zygotene-like spermatocytes (*n* = 336) (Figure 3P–R). Previous reports could not find evidence of H1t incorporation in *Dmc1*^-/-^ spermatocytes [54]. However, we found that similar to *Spo11* mutants, many zygotene-like spermatocytes in *Dmc1*^-/-^ mice (77.29%, *n* = 303) display a labelling with this histone (Figure 4S–U), although it is always very faint and never reaches wild type levels.

Mutant mice lacking HOP2 protein do not repair DSBs properly, since they fail to trigger the strand invasion process [64]. Regarding progression of meiosis, the phenotype of *Hop2*^-/-^ mice is then almost identical to that from *Dmc1*^-/-^, as *Hop2*^-/-^ spermatocytes are arrested during early stages before stable synapsis can occur. Our observations on this mutant indicate that the localization pattern of the markers analyzed did not significantly differ from those on *Dmc1* knockout mice (Supplementary Figure S2).

### 3.2. Epigenetic Markers in Synapsis-Defective Mutants

**Figure 5:**
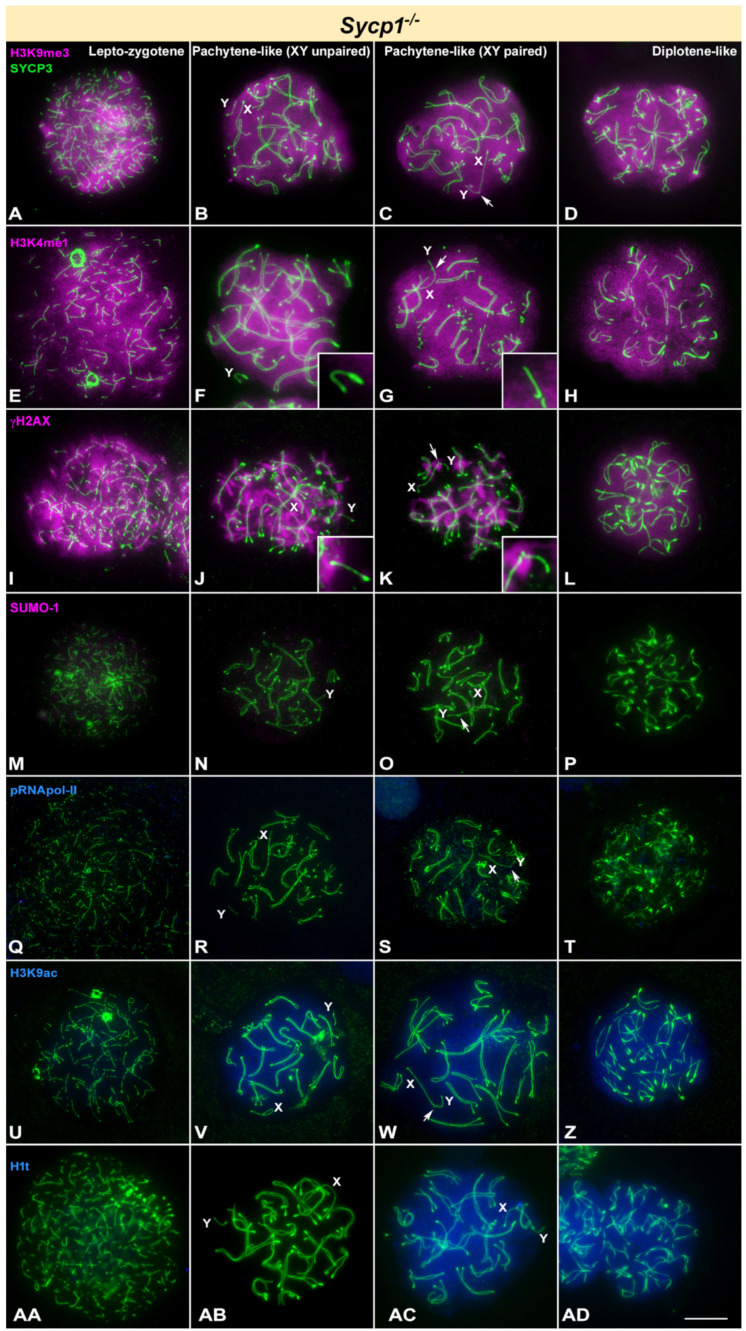
Immunolocalization of SYCP3 (green) and epigenetic markers of silencing (pink) or transcription activation (blue) in *Sycp1* knockout mice. X and Y indicate the sex chromosomes, which are identified by their size and the morphology of the AEs. Cells assigned to columns correspond to lepto-zygotene, pachytene-like with unpaired XY chromosomes, pachytene-like with paired XY (PAR indicated by arrows) and diplotene-like stages. (**A**–**D**). H3K9me3 seems visible all over the chromatin from leptotene-zygotene until diplotene and is slightly more concentrated around centromeres. X and Y chromosomes show the same labelling than the autosomes with no brighter regions. (**E**–**H**). A bright signal for H3K4me1 is observed in the whole nucleus from leptotene-zygotene (**E**). The Y chromosome (enlarged in the insets) is distinguishable at pachytene-like. It seems to be devoid of labelling when not paired with the X chromosome (**F**). Even when paired with the X chromosome (**G**), the Y chromosome shows a fainter labelling. H3K4me1 is brightly detected in the whole nucleus during diplotene-like stage (**H**). (**I**–**L**). γH2AX labels all chromosomes during lepto-zygotene (**I**), with the exception of the Y chromosome (inset). At pachytene-like (**J**), (**K**) labelling of γH2AX appears as large foci on the autosomes. The still unpaired Y chromosome shows a distal mark of the histone that presumably corresponds to the PAR (inset). Even when the X and Y chromosomes are paired, γH2AX signal is mostly concentrated around their paired region. Diplotene-like spermatocytes (**L**) show a less intense mark for the protein, but globally covering the nucleus. (**M**–**P**). SUMO-1 is not detected at any stage in this mutant, in agreement with its failure in XY body formation. (**Q**–**T**). No signal of pRNApol-II is detected at any stage. (**U**–**Z**). Localization pattern of H3K9ac. A very faint signal is observed in early stages (**U**). A more intense H3K9ac signal is already detected in pachytene-like (**V**–**W**) and diplotene stages (**Z**). Sex chromosomes are not excluded from this labelling. (**AA**–**AD**). H1t labelling is not detected in leptotene (**AA**). Some pachytene-like spermatocytes are devoid of labelling (**AB**), while others show an intense signal (**AC**). Diplotene-like (**AD**) spermatocytes show an intense H1t labelling. Scale bar: 10 µm.

The meiotic phenotype of *Sycp1*^-/-^ mice is characterized by the absence of a tripartite SC, lacking TFs and the central element of the SC [57]. In these mutants, AEs of homologous chromosomes form normally at leptotene (Figure 5A). In later stages AEs align side by side but appear physically separated [57] (Figure 5). Thus, from zygotene onwards we classified spermatocytes as pachytene-like and diplotene-like stages. In pachytene-like, we found that sex chromosomes may appear separated from each other, with no contact between them (Figure 5B) or paired at the PAR ends (Figure 5C) as in normal meiosis. A diplotene-like stage was defined by shorter AEs that usually show irregular thickened regions. Even though chromosome morphology in these mutants is reminiscent to that of wild type until a diplotene-like stage, our results indicate a clearly uncoupled progression of epigenetic events. All spermatocytes show a pattern of H3K9me3 localization that corresponds to wild type leptotene or zygotene, covering the whole nucleus. No specific accumulation in the sex chromosomes was detected at any stage (Figure 5A–D). A similar pattern is observed for H3K4me1, as this protein decorates the entire nucleus in all stages (Figure 5E–H). Extensive localization of γH2AX in the nucleus of *Sycp1*^-/-^ spermatocytes resembles that of wild type during early stages (Figure 5I). At pachytene-like stage (Figure 5J,K), this histone is observed as large foci that emanate from chromosomes, again resembling the zygotene distribution in normal meiosis. At diplotene-like, γH2AX shows a homogeneous nuclear labelling (Figure 5L). Regarding sex chromosomes, γH2AX is present in the Y chromosome as a single focus either before pairing with the X or after they become associated, most probably located at the PAR. This pattern represents the typical situation at the transition between zygotene and pachytene in wild type, and it is the prelude of γH2AX signal extension to form the sex body. However, such extension over the X and Y chromosomes and the formation of a sex body never occur in *Sycp1*^-/-^ spermatocytes. This feature is also revealed by the absence of SUMO-1 labelling at all stages (Figure 5M–P). Phosphorylated RNA pol-II was not found to accumulate at any stage (Figure 5Q–T), indicating low transcriptional activity. In contrast, an intense H3K9ac labelling is observed in this mutant in pachytene and diplotene spermatocytes (Figure 5U–Z). As previously described, pachytene spermatocytes incorporate H1t histone in levels similar to wild type (Figure 5AA–AD) [57].

**Figure 6:**
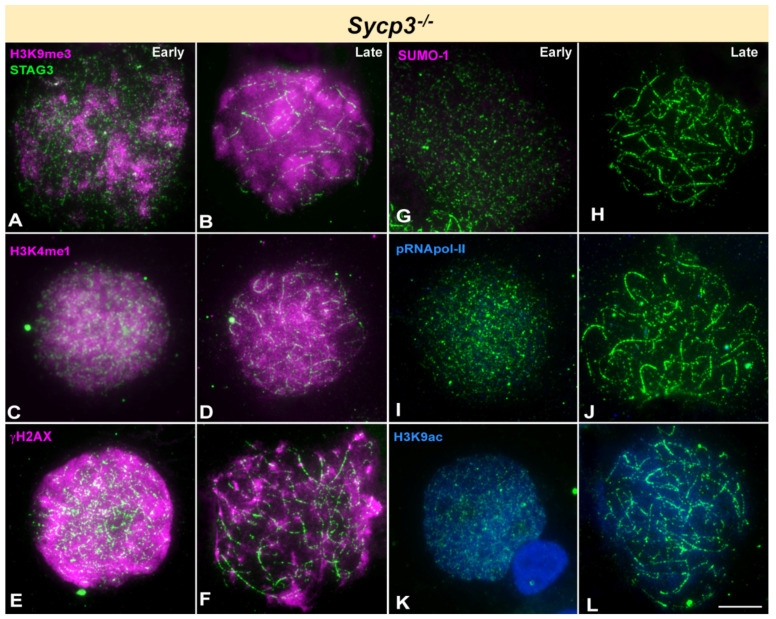
Immunolocalization of STAG3 (green) and epigenetic markers of silencing (pink) or transcription activation (blue) in *Sycp3* knockout mice. Cells have been divided into early or late stages. STAG3 appears in all stages as discontinuous or fragmented filaments, representing cohesin axes. (**A**,**B**). H3K9me3 distribution is not homogeneous in the nucleus in early stages (**A**) but it concentrates in some large areas. In mid stage (**B**) it appears more spread in the nucleus, with local accumulations lacking a clear relationship to specific chromosomal regions. (**C**,**D**). H3K4me1 covers almost the whole chromatin from early to late stages. (**E**,**F**). γH2AX localized to the whole nucleus in early stages. In late stages (**F**) it seems to concentrate in more discrete but large foci. (**G**,**H**). No SUMO-1 labelling is observed in spermatocytes at any stage. (**I**,**J**). No labelling of pRNApol-II is observed in any spermatocyte. (**K**,**L**). A faint signal of H3K9ac is detected in the nucleus in all stages. Scale bar: 10 µm.

To conclude the analysis of mutants with defects in SC formation, we studied *Sycp3*^-/-^ mice. Spermatocytes fail to form AEs in this model and they arrest very early during prophase-I [66]. Since SYCP3 could not be used as a cytological marker, we labelled spermatocytes with an anti-STAG3 antibody, which is a component of the cohesin complex and reveals the cohesin axis (CA) assembled along meiotic chromosomes (Figure 6) [78]. This staining can be used as a proxy to the localization of the axes of chromosomes. We just classified spermatocytes as early or late according to the extension of STAG3 filaments. Patches of H3K9me3 are seen from the very beginning of meiosis, when incipient CAs are formed, and remain visible until the latest spermatocytes found, presumably corresponding to the pericentromeric heterochromatin (Figure 6A,B). H3K4me1 labelling marks the entire nucleus during all stages and no other significant feature or difference between them is observed (Figure 6C,D). γH2AX is detected covering prominently the whole nucleus in the early stages and in a somehow restricted fashion in the latest stages observed (Figure 6E,F). SUMO-1, in turn, is completely absent in these spermatocytes (Figure 6G,H). pRNA pol-II is never detected in these cells (Figure 6I,J) and H3K9ac staining shows a faint signal dispersed over the whole chromatin that does not change from the earliest to the latest stages (Figure 6K,L). Finally, we could not find any evidences of H1t incorporation.

## 4. Discussion

The study of prophase-I progression in mammals has been traditionally focused on the analysis of chromosome synapsis, cohesion and recombination, and their regulatory factors. However, in the last few years the study of three-dimensional organization of chromatin by Hi-C techniques [20,21,22], the characterization of gene expression profiles by microarrays or RNA-seq [26,27,28,79] and the analysis of epigenetic features encompassed throughout prophase-I [31,35,36,37,80] have revealed meiosis as a multi-layered process, in which progression of synapsis and recombination is accompanied by changes in other processes. Consistently, it has been shown that mutating genes involved in synapsis, cohesion or recombination has a clear impact in chromatin organization. One of the most conspicuous effects of these mutations is the interference with MSCI, which has been reported in a wide range of studies [54,55,56,57,58,59]. A few studies have also revealed a general disruption of the normal patterns of gene expression in specific mutants (for instance, *Spo11* [81], *SMC1*β [56] and *Prdm9* [62]), but the massive use of transcriptomic analysis is increasing the bulk of knowledge in this specific field [30]. Interestingly, these studies have revealed that both autosomal and sex chromosomal genes are dysregulated and point to the uncoupling of gene expression and the morphological progression of meiosis [62]. Our results add new evidence to such uncoupling by showing the differential distortion of the epigenetic program and transcriptional activity in a variety of recombination and synapsis mutants, and provide a cytological framework to understand these alterations.

### 4.1. Epigenetic Signatures of Spermatocytes Change along Prophase-I

Our present results and previous studies [31,36,37] indicate that mammalian spermatocytes initiate meiosis, both in wild type and the mutants analyzed, with a defined set of epigenetic marks. This includes a broad nuclear distribution of H3K9me3 and H3K4me1. In contrast with the accumulation of γH2AX, the presence of these marks is not dependent on the induction of DSBs, as they appear also in *Spo11*^-/-^. Likewise, their presence in *Sycp3*^-/-^ mice indicates that this is not dependent on the initiation of SC formation. Moreover, the presence of these histone modifications at the beginning of meiosis, at least H3K9me3, seems critical for a proper meiosis outcome, as ablation of the methyltransferase SETDB1, which catalyzes H3K9 trimethylation, strongly hampering meiosis progression [43,47]. Both H3K9me3 and H3K4me1 histone modifications are related to transcription repression and heterochromatin formation [74,75,82,83], and their broad presence in the nucleus at leptotene could indicate that spermatocytes enter meiosis in a partially inactive state. Accordingly, the levels of pRNA pol-II and H3K9ac, which are indicative of transcription activity, are very low or undetectable at the beginning of meiosis. This does not mean that transcription is completely abolished. Recent studies have corroborated the expression of genes during leptotene and zygotene [27]. Moreover, the location pattern of these histone modifications is in contrast with the partial demethylation of DNA in early prophase-I, which could be related to a more permissive transcriptional state of the chromatin [84]. These contrasting facts can be reconciled by considering that the combination of these modifications, and potentially others, could result in a unique and precisely regulated epigenetic landscape at the beginning of meiosis, which leads to the expression of a specific set of genes and non-coding RNAs [27,30]. Regardless, in normal meiosis the epigenetic landscape changes with prophase-I progression: H3K9me3 and H3K4me fade away while pRNA pol-II and H3K9ac rise at mid pachytene. This could create the conditions for the change in the gene expression profile that occurs at pachytene [26,27,79], and further epigenetic transitions such as the replacement of histone H1 by the testis specific H1t [32,33]. Our analysis and previous reports reveal that spermatocytes are unable to complete this pachytene epigenetic transition in many recombination and synapsis knockout models [54].

### 4.2. Early Epigenetic Signatures Are not Lost in Most Recombination and Synapsis Mutants and Overlap with Late Signatures

**Figure 7:**
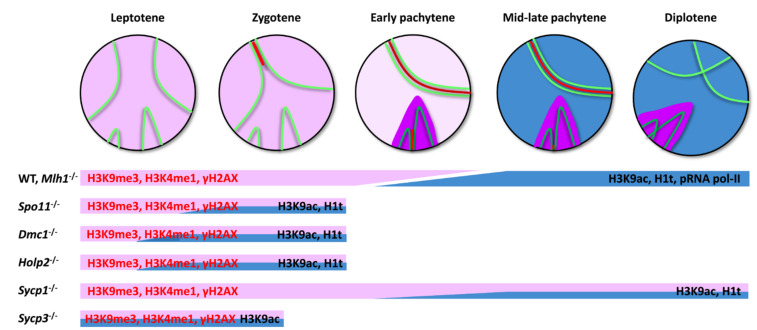
Schematic representation of the dynamics of the different epigenetic markers studied and their distribution in the different mouse models. Spermatocytes in the top row represent the progression of synapsis between homologous chromosomes or the sex chromosomes. AEs are represented in green and TFs in red. The chromatin of the depicted chromosomes is stained in pink for the early epigenetic pattern (representing mostly repressive marks) and is subsequently substituted for late epigenetic marks in pale blue (representing mostly permissive marks). The transition is produced between early (with chromatin in pale pink) and mid pachytene. Sex chromosomes display a particular pattern: they behave as autosomes in leptotene and zygotene but differ from early pachytene onwards in their accumulation of specific marks involved in MSCI. The composition of the sex body changes throughout pachytene and diplotene (represented as darker pink tones as MSCI is initiated). Each mouse model shows a specific pattern of progress in relation to synapsis and the appearance of epigenetic marks in the autosomes (represented as colored bars). Early (orange) and late (blue) patterns overlap in all mutant models. Sex chromosomes remain in a zygotene-like stage in all mutants (not represented).

With the exception of *Mlh1*^-/-^ mice, which complete all the epigenetic transitions analyzed, all other mutants studied here retain most or all the epigenetic features of leptotene and zygotene spermatocytes (Figure 7). This is not surprising considering that spermatocytes in *Spo11*^-/-^, *Dmc1*^-/-^, *Hop2*^-/-^, and *Sycp3*^-/-^ mice can only advance up to a zygotene-like stage in terms of synapsis and DNA repair. These cells are subsequently eliminated owing to the action of the so-called pachytene checkpoint, which eliminates spermatocytes at the stage IV of the seminiferous epithelium [54,60]. Accordingly, spermatocytes retain the early chromatin patterns and only show some signs of transition regarding the accumulation of H3K9ac. However, it is somehow unexpected that *Sycp1*^-/-^ mice are also unable to acquire a typical pachytene pattern. Although DNA repair is partially compromised in this mutant, it is capable of achieving homologous pairing and progress until a diplotene-like stage in terms of AE/LEs morphology. Moreover, some spermatocytes are able to reach a metaphase-I-like stage [57]. However, neither H3K9me3, H3K4me nor γH2AX are efficiently removed from these spermatocytes and these cells retain a zygotene pattern even in the most advanced stages. Strikingly, the preservation of early epigenetic marks is compatible with the incorporation of some late epigenetic ones, such as histone K3K9ac and H1t, indicating that such incorporation may occur in the presence of unresolved DSBs or severe synaptic defects. This is observed not only in *Sycp1*^-/-^ mice [57], but interestingly also in *Spo11*^-/-^ [54] and *Dmc1*^-/-^. Finally, as suggested by the immunostaining assay, normal pachytene RNA pol-II levels are not reached in those mutants.

The comparison of different mutants performed in this study illustrates the uncoupling between recombination, synapsis, and epigenetics, which can advance to different endpoints in relation to each other (Figure 7). This has interesting implications. On the one hand, the complex combination of epigenetic features in each mutant indicates that using single epigenetic marks such as H1t to assess the progression of spermatocytes throughout meiosis can be misleading. On the other hand, the coexistence of early and late epigenetic marks may explain the findings in *Prdm9*^-/-^ mice, in which synapsis and recombination are halted at very early stages (leptotene-zygotene), while the program of gene expression remained unaltered and spermatocytes showed the typical pachytene-diplotene gene expression profile [62]. Moreover, it must be considered that each mutation can have differential effects over the progression of each meiotic phenomena. In this sense, analysis of gene expression profile in *Spo11*^-/-^ mice revealed that genes usually active at pachytene are clearly downregulated [81], indicating that interference of meiosis at different points of the recombination or synapsis pathways can have different outcomes in terms of this proposed uncoupling. With the increasing use of transcriptomic approaches, comparison of different mutants could reveal an unanticipated diversity in the modifications of gene expression profiles.

### 4.3. Epigenetic Progression and Inactivation of Sex Chromosomes

The inactivation of sex chromosomes is one of the hallmarks of mammalian male meiosis. The reasons and mechanism of this inactivation have been intensely studied in the last twenty years [31,42,50,71,85,86,87,88,89,90,91,92,93]. Current models indicate that extensive asynapsis of the heterologous regions of the X and Y chromosomes triggers the accumulation of DNA repair factors such as BRCA1, ATR, and MDC1 at the beginning of pachytene, which would induce the accumulation of γH2AX and other protein factors and RNAs over the sex chromosomes, leading to their inactivation [93]. According to this, genes on both the X and Y chromosomes can be expressed in early prophase-I and then silenced in pachytene. Although there is increasing evidence that many genes and microRNAs are able to escape MSCI during pachytene and diplotene [27,30,94], it has been demonstrated that the unprogrammed expression of *Zfy1/2* genes, which are normally silenced during pachytene, can halt meiosis progression. Indeed, this has been proposed to be a cause of meiosis breakdown in mouse mutants that fail to inactivate sex chromosomes [52,53]. As indicated above, most of those mutants, including *Spo11*^-/-^, *Dmc1*^-/-^, *Hop2*^-/-^, and *Sycp3*^-/-^, eliminate the spermatocytes in a stage that probably corresponds to early pachytene and that correlates to the stage IV of the mouse seminiferous epithelium [54,60].

All mutants analyzed in this work, except for *Mlh1*^-/-^, fail to perform a recognizable MSCI. However, they show differences in the epigenetic transitions reached by the sex chromosomes, particularly the Y chromosome. In *Spo11*^-/-^ mutants, owing to the absence of DSBs, sex chromosomes never incorporate γH2AX. Although a pseudo-sex body is formed and it incorporates some MSCI markers such as γH2AX, SUMO-1, and XMR, it is not related to sex chromosomes [54,55]. Contrarily, *Dmc1*^-/-^ and *Hop2*^-/-^ may proceed to a stage in which the Y chromosome incorporates γH2AX, but this histone mark is always restricted to one chromosome end and it never extends to the rest of the chromosome. The late incorporation of γH2AX is characteristic of the Y chromosome in normal meiosis and denotes the delayed production of DSBs in this chromosome at the zygotene-pachytene transition [31,95,96]. Our observations suggest that the induction of H2AX phosphorylation following DNA damage is functionally distinct from the phosphorylation that leads to MSCI in this chromosome. Notably, in *Sycp1*^-/-^ mice, although X and Y chromosomes undergo homologous pairing, γH2AX never extends specifically beyond the PAR, reinforcing the idea of the uncoupling between morphological and epigenetic progression.

All of these observations are compatible with an interference of MSCI initiation in spermatocytes from these, and probably other, knockout mice models. However, considering the interference of the overall epigenetic progression discussed above, it seems that at least some of these mutants are not properly performing all of the epigenetic transitions needed to progress into pachytene, one of which could be the initiation of the specific processes leading to MSCI. Therefore, those mutants could fail to initiate sex chromosome inactivation not only due to the fact that they are unable to trigger MSCI mechanisms, but also since they never reach the stage at which these events are initiated. In this sense, the defects attributed to a failure to inactivate sex chromosomes in these mutants should be placed in a context of general dysregulation of transcription in the whole nucleus, as recently suggested [62]. Whether dysregulation of transcription in autosomes -leading to the expression of genes potentially harmful for meiosis- is an important factor in the regulation of meiosis progression or just a side-consequence is an interesting question that could be explored in future studies. Under this view, the phenotype of many meiotic mutants can be re-evaluated to incorporate the epigenetic landscape and the desynchronization of meiotic events as an important factor to explain meiosis breakdown, together with defects in recombination, synapsis and MSCI. Clearly, additional factors such as changes in chromatin interactions [21] and regulation of gene expression profiles [56,62] should be added to this perspective.

## Data Availability

Not applicable.

## Supplementary Materials

The following are available online at https://www.mdpi.com/article/10.3390/cells10092311/s1, Figure S1: Immunolocalization of SYCP3, SUMO-1 and γH2AX at zygotene-like stage in Spo11 knockout mice. Figure S2: Immunolocalization of SYCP3 and epigenetic marks in Hop2 knockout mice.
