# Supplementary material for: Epigenetic Dysregulation of Mammalian Male Meiosis Caused by Interference of Recombination and Synapsis
Source: Cells. 2021 Sep 3;10(9):2311. doi: 10.3390/cells10092311 (PMC8467405; doi:10.3390/cells10092311)
Supplement: Supplementary file 1 [file cells-10-02311-s001.zip › cells-1307083-supplementary.pdf]

cells

MDPI

Article

Epigenetic Dysregulation of Mammalian Male Meiosis Caused by Interference of Recombination and Synapsis

Roberto de la Fuente [1,2], Florencia Pratto [3], Abrahan Hernández-Hernández [4,†], Marcia Manterola [5], Pablo López-Jiménez [1], Rocío Gómez [1], Alberto Viera [1], María Teresa Parra [1], Anna Kouznetsova [4], R. Daniel Camerini-Otero [3], and Jesús Page [1,*]

Supplementary Materials

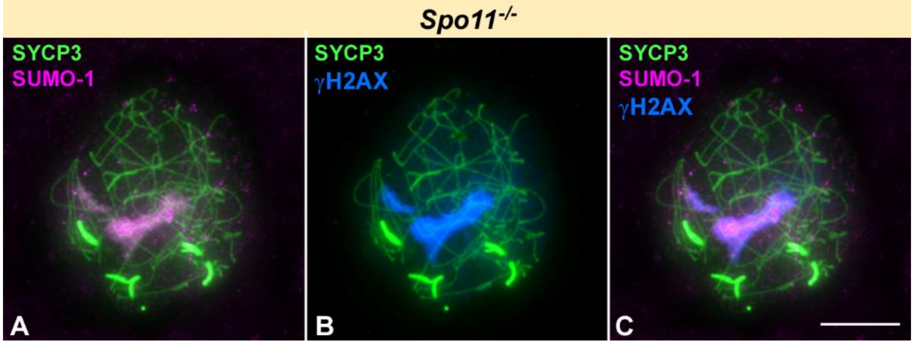

Supplementary Figure S1: Immunolocalisation of SYCP3 (green) SUMO-1 (pink) and γH2AX (blue) in a zygotene-like spermatocyte in *Spo11* knockout mice. A diffuse signal is observed with SUMO-1, which co-localised with the signal of γH2AX. Scale bar: 10 µm.

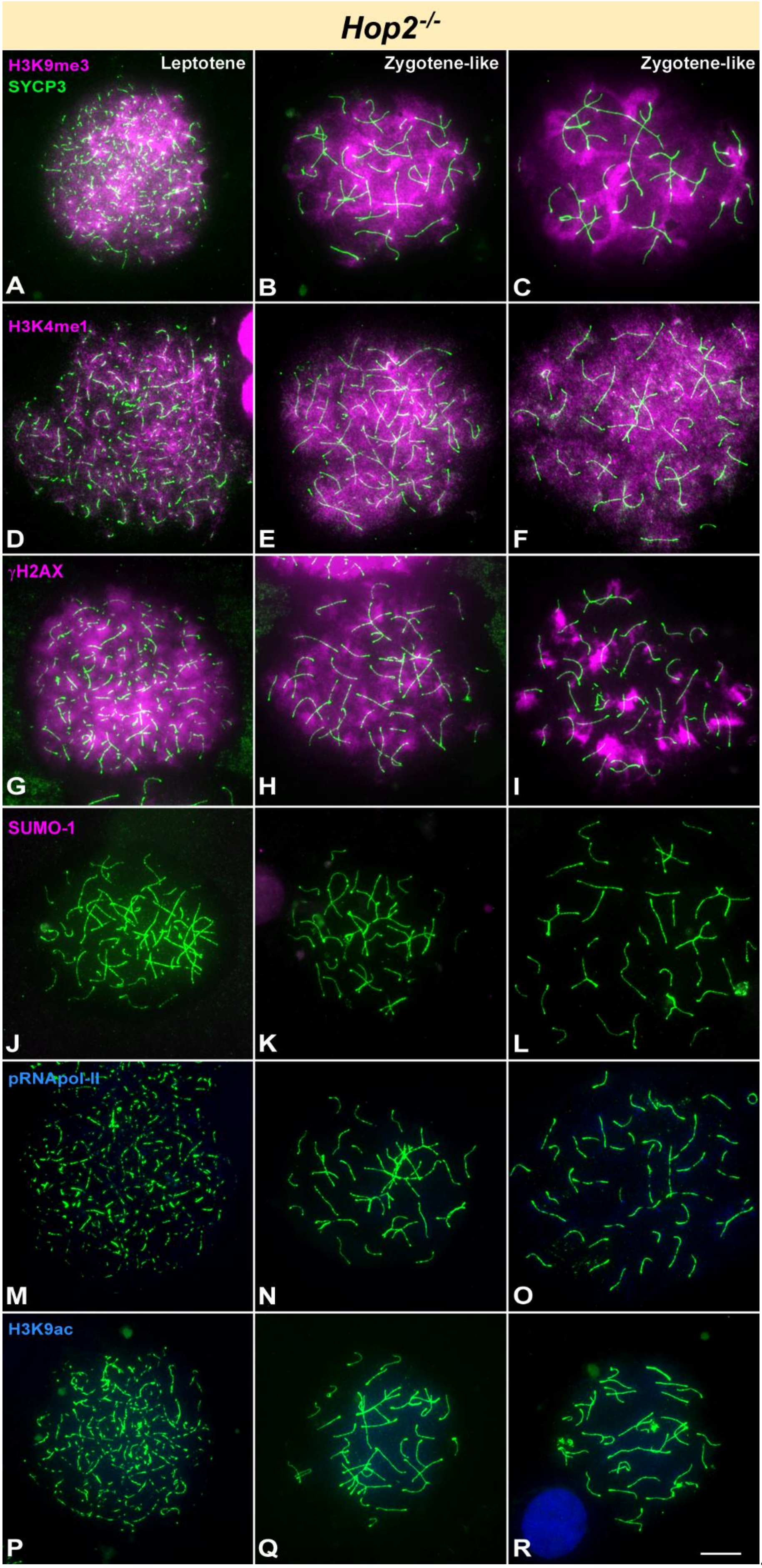

Hop2-/-
H3K9me3
SYCP3
Leptotene
Zygotene-like
Zygotene-like
A
B
C
H3K4me1
D
E
F
γH2AX
G
H
I
SUMO-1
J
K
L
pRNApol-II
M
N
O
H3K9ac
P
Q
R

Supplementary Figure S2. Immunolocalisation of SYCP3 (green) and epigenetic markers of silencing (pink) or transcription activation (blue) in *Hop2* knockout mice. Cells in each column are classified as leptotene or zygotene-like. (**A-C)**. An intense H3K9me3 labelling is detected in the nucleus in all stages, although it appears slightly more intense in the pericentromeric regions at the late stages. (**D**-**F**). H3K4me1 strongly marks the entire nucleus of the spermatocytes at all stages. (**G**-**I**). γH2AX labelling is spread over the entire nucleus in leptotene and some zygotene-like spermatocytes. In some zygotene-like stages (**I**) labelling concentrates as large foci over some autosomal regions. (**J**-**L**). SUMO-1 is not detected at any stage on these mutants. (**M**-**O**). No signal of pRNApol-II is detected at any stage. (**P**-**R**). H3K9ac is not detected in early leptotene. At zygotene-like, a weak signal is observed in the whole nucleus. Scale bar: 10 μm.
